# Poly(*m*-Phenylenediamine) Nanospheres and Nanorods: Selective Synthesis and Their Application for Multiplex Nucleic Acid Detection

**DOI:** 10.1371/journal.pone.0020569

**Published:** 2011-06-23

**Authors:** Yingwei Zhang, Hailong Li, Yonglan Luo, Xu Shi, Jingqi Tian, Xuping Sun

**Affiliations:** 1 State Key Lab of Electroanalytical Chemistry, Changchun Institute of Applied Chemistry, Changchun, Jilin, People's Republic of China; 2 Chinese Academy of Sciences, Graduate School of the Chinese Academy of Sciences, Beijing, People's Republic of China; 3 Institute of Virology and AIDS Research, First Affiliated Hospital, Jilin University, Changchun, Jilin, People's Republic of China; University of Pennsylvania, United States of America

## Abstract

In this paper, we demonstrate for the first time that poly(*m*-phenylenediamine) (PMPD) nanospheres and nanorods can be selectively synthesized via chemical oxidation polymerization of *m*-phenylenediamine (MPD) monomers using ammonium persulfate (APS) as an oxidant at room temperature. It suggests that the pH value plays a critical role in controlling the the morphology of the nanostructures and fast polymerization rate favors the anisotropic growth of PMPD under homogeneous nucleation condition. We further demonstrate that such PMPD nanostructures can be used as an effective fluorescent sensing platform for multiplex nucleic acid detection. A detection limit as low as 50 pM and a high selectivity down to single-base mismatch could be achieved. The fluorescence quenching is attributed to photoinduced electron transfer from nitrogen atom in PMPD to excited fluorophore. Most importantly, the successful use of this sensing platform in human blood serum system is also demonstrated.

## Introduction

During the past decades, conducting polymers (CPs) have constituted a subject of research for their unique properties and important application potential [1]. Polyaniline is one of the most studied CPs due to its chemical stability and relative high conductivity [2] and, at the same time, polymers based on aniline derivatives have also been widely investigated [3]. Among them, poly(phenylenediamine) (PPD) homopolymer is a highly aromatic polymer containing 2,3-diaminophenazine or quinoraline repeating unit and exhibiting high thermostability and has found important applications in sensor designing, immunospecies detection, and as component of rechargeable cells etc [4]–[13]. PPD is usually prepared by electrochemical [14] and chemical oxidation polymerization [15]. Although we [16] and other researchers [17]–[19] have successfully prepared poly(*o*-phenylenediamine) nanobelts and microparticles by chemical oxidation polymerization method, respectively, the selective synthesis of PPD with different morphologies has not been addressed so far.

On the other hand, it is vitally important to develop rapid, cost-effective, sensitive and specific methods for the detection of nucleic acid due to their various applications in gene expression profiling, clinical disease diagnostics and treatment [20]. The increasing availability of nanostructures has created widespread interest in their use in biotechnological system for diagnostic application [21]. Indeed, the use of a variety of nanostructures for this purpose has been well-demonstrated [22]. Recently, there have been many efforts toward developing homogeneous fluorescence assays based on fluorescence resonance energy transfer (FRET) or quenching mechanism for nucleic acid detection [23]. The use of nanostructures as a “nanoquencher” has a remarkable advantage in that the same nanostructure has the ability to quench dyes of different emission frequencies and thus the selection issue of a fluorophore-quencher pair is eliminated from the nanostructure-involved system [23], [24]. Up to now, a number of structures have been successfully used by us and other researchers in this assay, including gold nanoparticles, single-walled carbon nanotubes (SWCNTs), multi-walled carbon nanotubes, carbon nanoparticles, carbon nanospheres, nano-C_60_, mesoporous carbon microparticles, graphene oxide (GO), polyaniline nanofibres, poly(*o*-phenylenediamine) colloids, poly(2,3-diaminonaphthalene) microspheres, coordination polymer colloids and nanobelts, Ag@poly(*m*-phenylenediamine) core-shell nanoparticles, tetracyanoquinodimethane nanoparticles, and supramolecular microparticles [23]–[46]. For the SWCNT or GO system, it has drawbacks: (1) several hours' sonication is required to disperse SWCNT in an organic solvent like N,N-dimethylformamide (DMF) [30]; (2) the GO preparation by the Hummer's method is time-consuming and labor-intensive [47]. We have also found that conjugation polymer poly(*p*-phenylenediamine) nanobelts (PNs) can serve as an effective fluorescent sensing platform for multiplex nucleic acid detection [48]; however, this system still has two serious drawbacks which limit its practical use: (1) the nanobelts are tens of micrometers in length and thus tend to sink in aqueous solution due to the gravity, causing the problem of stability in detection; (2) it has poor discrimination ability in that it gives only 8.8% difference of detection signal between single-base mismatched and complementary sequences [48]. Accordingly, developing new nanostructure-based fluorescent sensing platform to overcome all these drawbacks is highly desired.

In this paper, we report on the selective synthesis of poly(*m*-phenylenediamine) (PMPD) nanospheres and nanorods by chemical oxidation polymerization of MPD monomers using ammonium persulfate (APS) as an oxidant at room temperature for the first time. It is found that the pH value is key to controlling the morphology of the nanostructures and fast polymerization rate favors the anisotropic growth of PMPD under homogeneous nucleation condition. We further demonstrate that such PMPD nanostructures can serve as an effective fluorescent sensing platform for multiplex nucleic acid detection. A detection limit as low as 50 pM and a high selectivity down to single-base mismatch could be achieved. The fluorescence quenching is attributed to photoinduced electron transfer from nitrogen atom in PMPD to excited fluorophore. Most importantly, the successful use of this sensing platform in human blood serum system is also demonstrated.

## Results and Discussion

**Figure 1:**
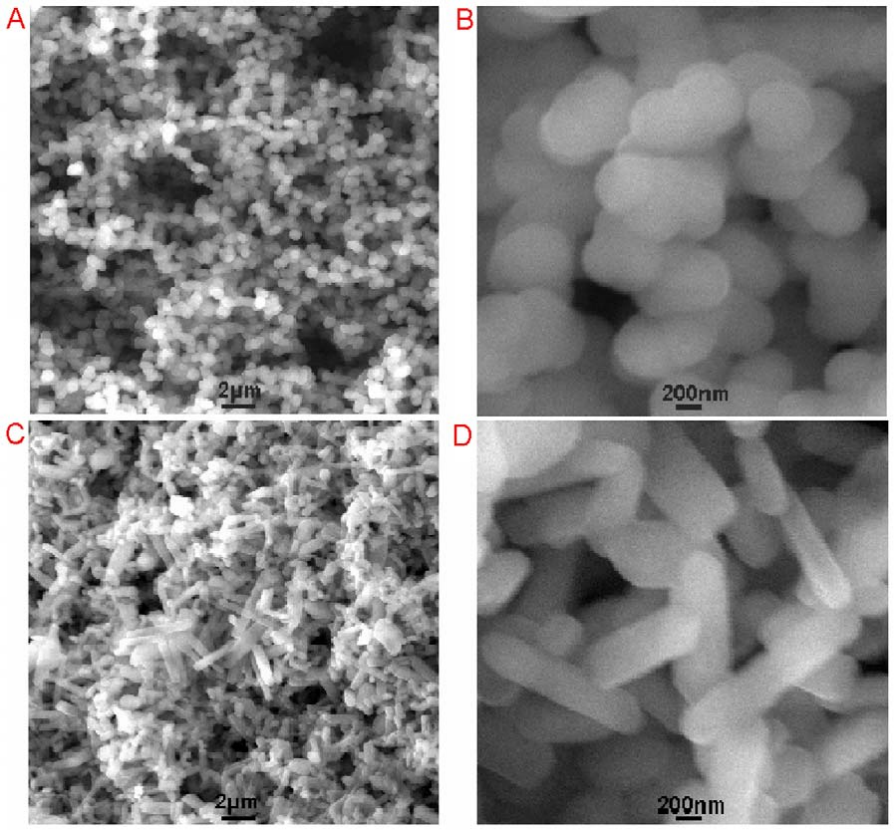
Instrumental analysis of the precipitate thus formed. Low magnification SEM images of the PMPD nanostructures formed using (A) water and (C) NMPD as reaction solvent, (B) and (D) corresponding to the high magnification SEM images.

Figure 1A shows low magnification SEM image of the products thus formed in water (sample 1, see **Materials and Methods** for preparation details), indicating that they consist exclusively of a large amount of nanoparticles. A close view of such nanoparticles further reveals that they are nearly spherical in shape and have size ranging from 300 to 600 nm, as shown in Figure 1B. The chemical composition of the nanospheres was determined by the energy-dispersed spectrum (EDS), as shown in Figure S1. The peaks of C and N elements are observed, indicating the nanospheres are formed from MPD. The presence of the peaks of S and O elements can be attributed to the fact that the polymerization of MPD by APS yields cationic polymer PMPD due to the proton doping effect, the SO_4_^2−^ (the reduced product of APS) and excessive S_2_O_8_^2−^ as counter-ions, however, will diffuse into the PMPD nanostructures for charge compensation [49], [50]. Very interestingly, it is found that the PMPD morphology can be changed by simply varying the reaction solvent used. Figure 1C and 1D shows typical SEM images of the products obtained with the use of N-methylpyrrolidone (NMPD) as reaction solvent, under otherwise identical conditions used for preparing sample 1. It is clearly seen that a large quantity of nanorods are produced as the main products. It was found that the use of N,N-dimethylformamide (DMF) and ethanol as the reaction solvent lead to nanorods (Figure S2A) and nanoshperes (Figure S2B), respectively. The possible mechanism of the effect of solvent in controlling the PMPD morphology is proposed as follows: The polymerization of MPD monomers by APS leads to a decrease of pH value of the system. Given the weak basic nature of NMPD and DMF, they are expected to neutralize the protons generated and thus the rate of polymerization of MPD is accelerated, which may favor the anisotropic growth of PMPD under homogeneous nucleation condition [51], [52]. It was found that polymerization of MPD monomers using water as reaction solvent but at basic condition also produced rod-like products. (Figure S2C). All these observations indicate that the pH value has played a critical role in controlling the morphology of the nanostructures. It is important to mention that these PMPD nanospheres and nanorods have smaller sizes and higher zeta potential (5 mV) and thus their dispersion exhibits better stability than PNs [48]. Indeed, we have found that such PMPD nanostructures are well-dispersed in water or buffer solutions. The resultant dispersions are very stable and no precipitation is observed within a couple of days.

**Figure 2:**
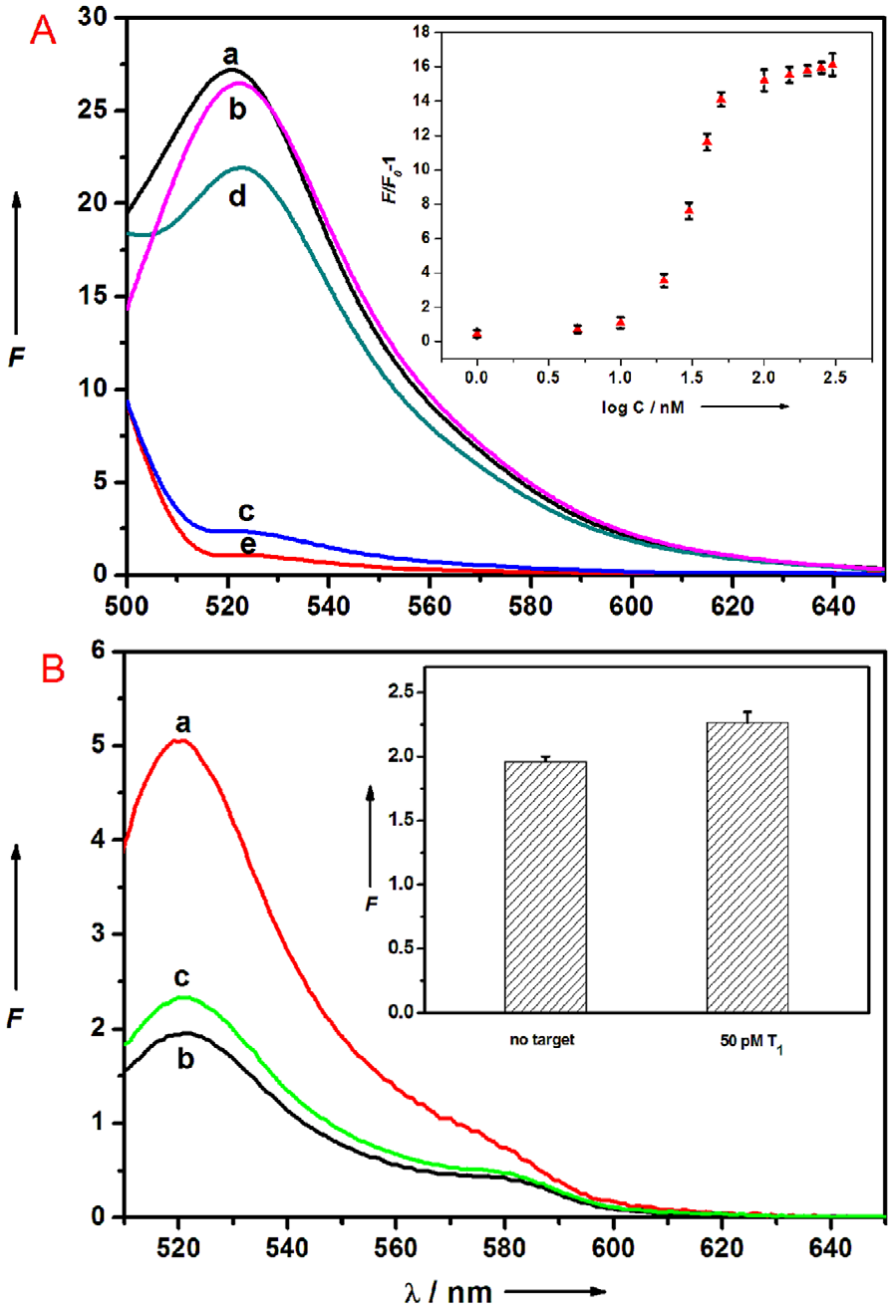
Performance of target DNA detection and determination of detection limit. (A) Fluorescence emission spectra of P_HIV_ (50 nM) at different conditions: (a) P_HIV_; (b) P_HIV_+300 nM T_1_; (c) P_HIV_+PMPD nanorods; (d) P_HIV_+PMPD nanorods+300 nM T_1_. Curve e is the emission spectra of PMPD nanorods. Inset: fluorescence intensity change (*F*/*F*_0_–1) of P_HIV_–PMPD nanorods complex (where *F*_0_ and *F* are the fluorescence intensity without and with the presence of T_1_, respectively) plotted against the logarithm of the concentration of T_1_. (B) (a) Fluorescence emission spectra of P_HIV_ (500 pM), (b) fluorescence quenching of P_HIV_ (500 pM) by 1-µL PMPD nanorods, and (c) fluorescence recovery of (b) by T_1_ (50 pM). Inset in Figure 2B: the corresponding fluorescence intensity histograms with error bar. Excitation was at 480 nm, and the emission was monitored at 522 nm. All measurements were done in Tris-HCl buffer in the presence of 5 mM Mg^2+^ (pH: 7.4). 10-µL PMPD nanorods were used in each measurement.

To test the feasibility of using PMPD nanostructures as an effective fluorescent sensing platform for nucleic acid detection, we chose PMPD nanorods and an oligonucleotide sequence associated with human immunodeficiency virus (HIV) as a model system. Figure 2A shows the fluorescence emission spectra of P_HIV_, the FAM-labeled probe, at different conditions. In the absence of PMPD, the fluorescence spectrum of P_HIV_ exhibits strong fluorescence emission due to the presence of the fluorescein-based dye (curve a). However, the presence of PMPD results in about 96% quenching of the fluorescence emission (curve c), revealing that PMPD can strongly adsorb ssDNA and quench the fluorescent dye very effectively. However, the P_HIV_–PMPD complex exhibits significant fluorescence enhancement upon its incubation with complementary target T_1_ over a 1-h period, leading to a 77% fluorescence recovery (curve d). Note that the fluorescence of the free P_HIV_ was, however, scarcely influenced by the addition of T_1_ in the absence of PMPD (curve b). It should be mentioned that the PMPD sample exhibits weak fluorescence emission (curve e) which contributes a little to the whole fluorescence intensity of each sample measured. Hence, a background fluorescence subtraction is performed for all PMPD-involved measurements. Figure 2A inset illustrates the fluorescence intensity changes (*F*/*F*_0_–1) of P_HIV_–PMPD complex upon addition of different concentrations of T_1_, where *F*_0_ and *F* are FAM fluorescence intensities at 522 nm in the absence and presence of T_1_, respectively. In the DNA concentration range of 5–300 nM, a dramatic increase of FAM fluorescence intensity was observed, which suggests that the nanorod/DNA assembly approach is effective in probing biomolecular interactions due to the excellent signalling process. It is worthwhile mentioning that optimal signal-to-noise ratio of 3.8∶1 and thus low detection limit can be achieved by decreasing the amount of PMPD and P_HIV_ used. A detection limit as low as 50 pM can be achieved when 1-µL PMPD sample and 500 pM P_HIV_ are used in this system (Figure 2B).

**Figure 3:**
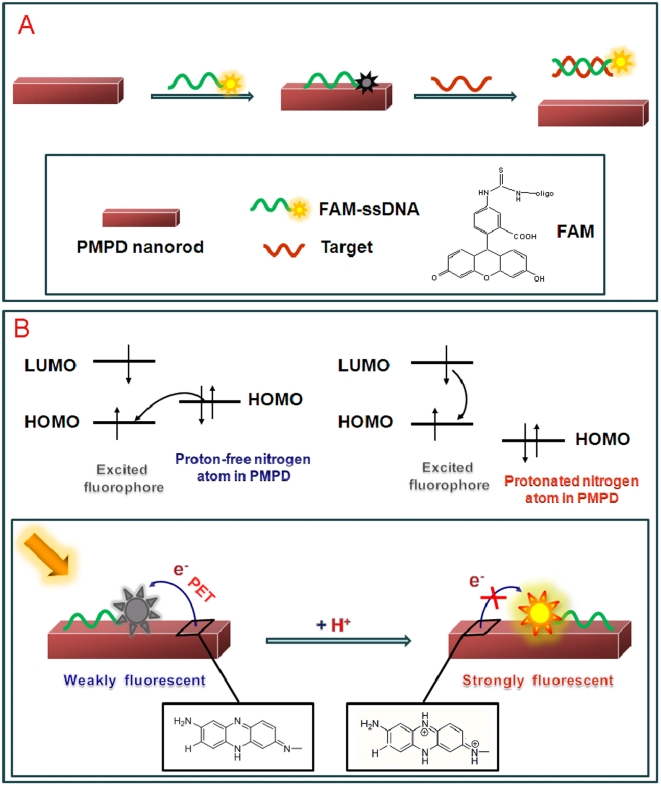
Illustration of the sensing process and fluorescence quenching mechanism. (A) A schematic (not to scale) illustrating the fluorescence-enhanced nucleic acid detection using PMPD nanorod as a sensing platform and (B) a schematic illustrating the photo-induced electron transfer (PET) process to explain the mechanism of fluorescence quenching.

**Figure 4:**
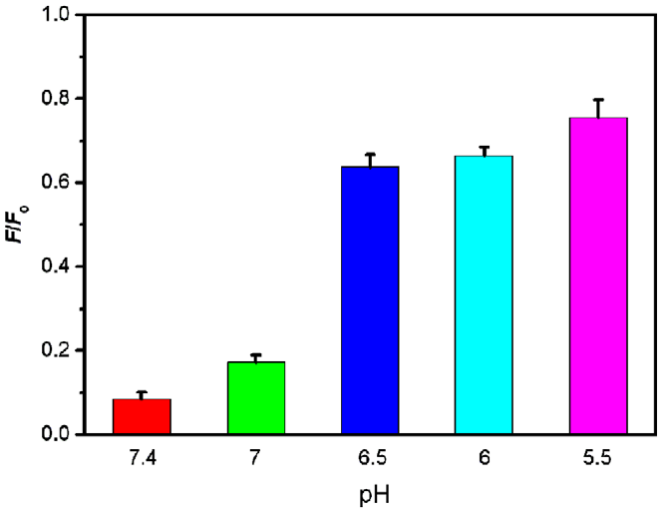
Influence of pH value on the fluorescence quenching. Fluorescence quenching histograms of of P_HIV_ (50 nM) by PMPD nanorods at different pH values (where *F* and *F*_0_ are the fluorescence intensity with and without the presence of PMPD). 10-µL PMPD nanorods were used in each measurement.

Because PMPD is a π-rich polymer, it can strongly and effectively adsorb single-stranded DNA (ssDNA) on its surface via π-π stacking between unpaired DNA bases and PMPD [53]. The zeta potential of the nanorods was measured to be about 5 mV in water, indicating that the nanorod has a low positively charged surface. However, the electrostatic attractive interactions between nanorod and negatively charged backbone of ssDNA contribute little to the adsorption of ssDNA on nanorod in the presence of a large amount of salt in buffer [44]. In contrast, the PMPD nanorod should have weak or even no binding with double-stranded DNA (dsDNA) due to the absence of unpaired DNA bases and the rigid conformation of dsDNA. Figure 3A presents a schematic to illustrate the fluorescence-enhanced nucleic acid detection using PMPD nanorod as a sensing platform. The DNA detection is accomplished by the following two steps: (1) PMPD binds dye-labeled ssDNA and quenches the fluorescence of the dye when they are brought into close proximity. (2) The subsequent hybridization of the probe with its target produces dsDNA which detaches from PMPD, leading to fluorescence recovery. The release of the resultant dsDNA from PMPD can be supported by the following experimental observation that there is no obvious fluorescence change observed after the removal of the PMPD nanorods from the hybridized solution by centrifugation, as shown in Figure S3. The observed fluorescence quenching in our present study could be attributed to photoinduced electron transfer (PET) from nitrogen atom in PMPD to excited fluorophore FAM when they are brought into close proximity [54]. Figure 3B illustrates the quenching mechanism involved. When the fluorophore FAM is excited, an electron from the highest occupied molecular orbital (HOMO) is promoted to the lowest unoccupied molecular orbital (LUMO), leaving an electronic vacancy in the fluorophore HOMO, which is filled by transfer of an electron from the higher energy HOMO of the nitrogen atom in PMPD serving as a donor. The overall effect of PET process is that the excited state life time is shortened and little fluorescence occurs, leading to fluorescence quenching. Upon protonation of the donor, however, the redox potential of the protonated donor is raised and its HOMO becomes lower in energy than that of the fluorophore. Consequently, electron transfer is hindered and fluorescence quenching is suppressed. This PET-based fluorescence quenching is confirmed by the experimental observation that the quenching is suppressed with the decrease of pH value and thus the increase of protonation degree of donor [18], as shown in Figure 4.

It should be noted that the amount of PMPD nanorods used in this system has profound effect on the efficiency of the fluorescence quenching and the subsequent recovery. Figure S4 shows the fluorescence intensity histograms of five samples with the use of 0, 5, 10, 15, and 20-µL PMPD nanorods sample, respectively. It suggested that the use of increased amount of nanorods leads to an increase in quenching efficiency but a decrease in recovery efficiency. The above observations can be reasoned as follows: When the ssDNA probe molecules are mixed with nanorods, they will adsorb on the nanorod surface. Obviously, the use of more nanorods leads to more efficient adsorption of ssDNA and thus higher quenching efficiency. But at the same time, the possibility of direct surface adsorption of target molecules on those excess nanorods increases during the following hybridization process. As a result, decreased hybridization efficiency and thus lower recovery efficiency is observed. Based on these observations, an optimal volume of 10 µL was chosen in our present study if not specified. Figure S5 shows a Stern–Volmer quenching curve describing *F_0_*/*F* as a function of MPD concentration, where *F*_0_ and *F* are FAM fluorescence intensities at 522 nm in the absence and the presence of PMPD nanorods, respectively. The plot is linear in the concentration range of 0 to 5 µM and the Stern–Volmer quenching constant (K_SV_) is calculated to be 3.768×10^5^ M^−1^ [55].

**Figure 5:**
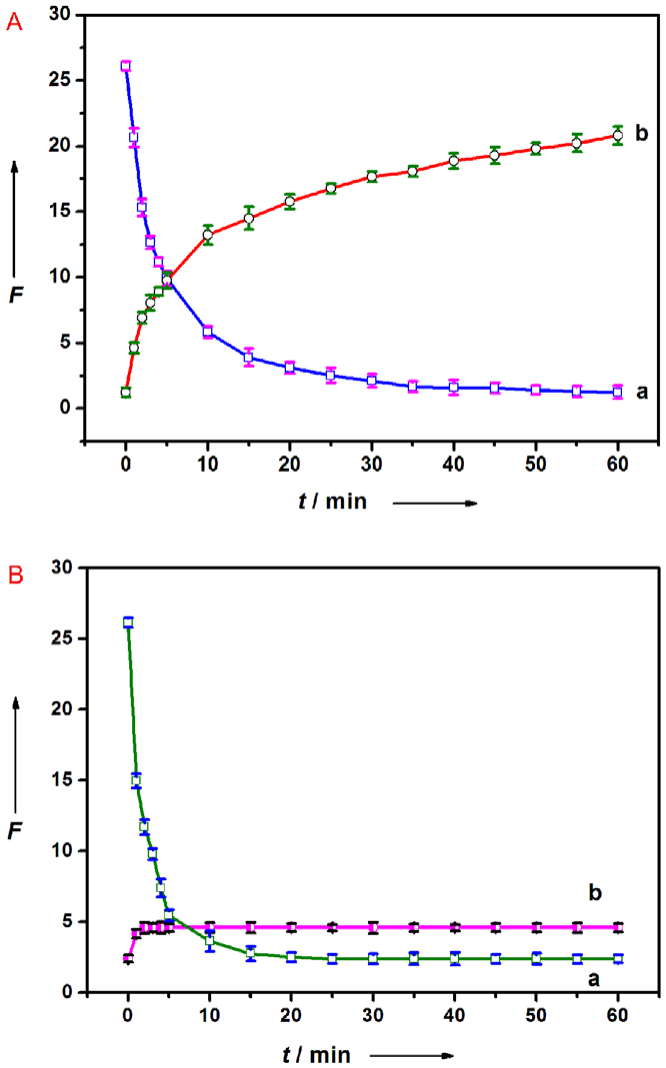
Kinetic behaviour study of fluorescence quenching and recovery at different temperatures. (a) Fluorescence quenching of P_HIV_ (50 nM) by PMPD nanorods and (b) fluorescence recovery of P_HIV_–PMPD by T_1_ (300 nM) as a function of incubation time (A: 25°C, B: 50°C). Excitation was at 480 nm, and the emission was monitored at 522 nm. All measurements were done in Tris-HCl buffer in the presence of 5 mM Mg^2+^ (pH: 7.4). 10-µL PMPD nanorods were used in each measurement.

The kinetic behaviors of P_HIV_ and PMPD, as well as of the P_HIV_–PMPD complex incubated with T_1_, were also studied by collecting the time-dependent fluorescence emission spectra. Curve a in Figure 5A shows the fluorescence quenching of P_HIV_ in the presence of PMPD as a function of incubation time at room temperature of 25°C. In the absence of the target, the curve exhibits a rapid reduction in the first 20 min and a slow decrease over a period of 40 min. Curve b in Figure 5A shows the subsequent fluorescence recovery of P_HIV_–PMPD by T_1_ in Tris-HCl buffer as a function of incubation time. In the presence of the target T_1_, the curve shows a fast increase in the first 10 min, followed by a slow process over a period of 50 min. The best fluorescence response was obtained after 1 h of incubation time. All above observations indicate that both ssDNA–PMPD association and dsDNA–PMPD dissociation occur faster than SWCNT but slower than GO system [24], [30], [36], [37]. These results are quite similar to those obtained from PN system [48]. We also investigated the influence of temperature on the kinetic behaviors of these two processes. Figure 5B shows the corresponding results obtained at 50°C, indicating that the time required to reach equilibrium is greatly shortened for both the quenching and the subsequent recovery process. It should be noted the decrease of fluorescence recovery in intensity at elevated temperature is observed in our present study, which can be attributed to that hybridization temperature close to the melting temperature does not favor duplex formation [56], leading to decreased hybridization and thus decreased fluorescence recovery efficiency.

**Figure 6:**
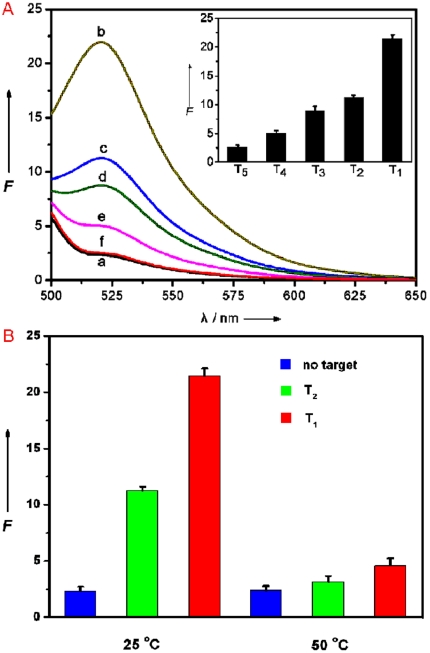
Evaluation of discrimination ability at different temperatures. (A) Fluorescence emission spectra of P_HIV_ (50 nM) at different conditions: (a) P_HIV_–PMPD complex; (b) P_HIV_–PMPD complex+300 nM T_1_; (c) P_HIV_–PMPD complex+300 nM T_2_; (d) P_HIV_–PMPD complex+300 nM T_3_; (e) P_HIV_–PMPD complex+300 nM T_4_; (f) P_HIV_–PMPD complex+300 nM T_5_. Inset: fluorescence intensity histogram with error bar. (B) Fluorescence signal enhancement of P_HIV_–PMPD complex upon incubation with 300 nM T_1_ and 300 nM T_2_ at 25 and 50°C, respectively. 10-µL PMPD nanorods were used in each measurement.

It is worthwhile mentioning that this sensing platform can well differentiate complementary and mismatched sequences. Figure 6A shows the fluorescence responses of P_HIV_–PMPD complex toward complementary target T_1_, single, two, and three-base mismatched target (T_2_, T_3_, and T_4_, respectively). The fluorescence intensity change (*F*/*F*_0_–1) value obtained upon addition of 300 nM of T_2_, T_3_, and T_4_ is about 46%, 33%, and 14% of the value obtained upon addition of 300 nM of T_1_ into P_HIV_–PMPD complex at room temperature of 25°C, respectively (where *F*_0_ and *F* are the fluorescence intensity without and with the presence of target). However, the addition of a non-complementary target to P_HIV_ (T_5_) leads to no observable fluorescence recovery (curve f). Compared to the complementary target, the mismatched target should have lower hybridization ability toward the adsorbed dye-labeled ssDNA probe, leading to a decreased hybridization and thus decreased fluorescence recovery efficiency. The inset is the corresponding fluorescence intensity histograms with error bar. It is worthwhile mentioning that, for the PN system, the addition of 300 nM of T_2_ only leads to a 8.8% difference between complementary and single-base mismatched target under otherwise identical conditions [48], but our new platform described herein gives a 49% difference which is about 5.6 times that of PN system, suggesting this sensing platform can greatly improve discrimination ability. We also performed hybridization experiments at an elevated temperature of 50°C and compared the fluorescence signal enhancement of P_HIV_–PMPD complex upon incubation with T_1_ and T_2_ at 25 and 50°C, respectively, as shown in Figure 6B. We found that the fluorescence intensity change (*F*/*F*_0_–1) value obtained upon addition of T_2_ is about 33% of the value obtained upon addition of T_1_ into P_HIV_–PMPD complex at 50°C. All the above observations indicate that the present nucleic acid detection system can distinguish complementary and mismatched nucleic acid sequences and its discriminating ability increases with increased temperature which makes the hybridization harder for probe and mismatched target due to that hybridization temperature close to the melting temperature does not favour duplex formation.

**Figure 7:**
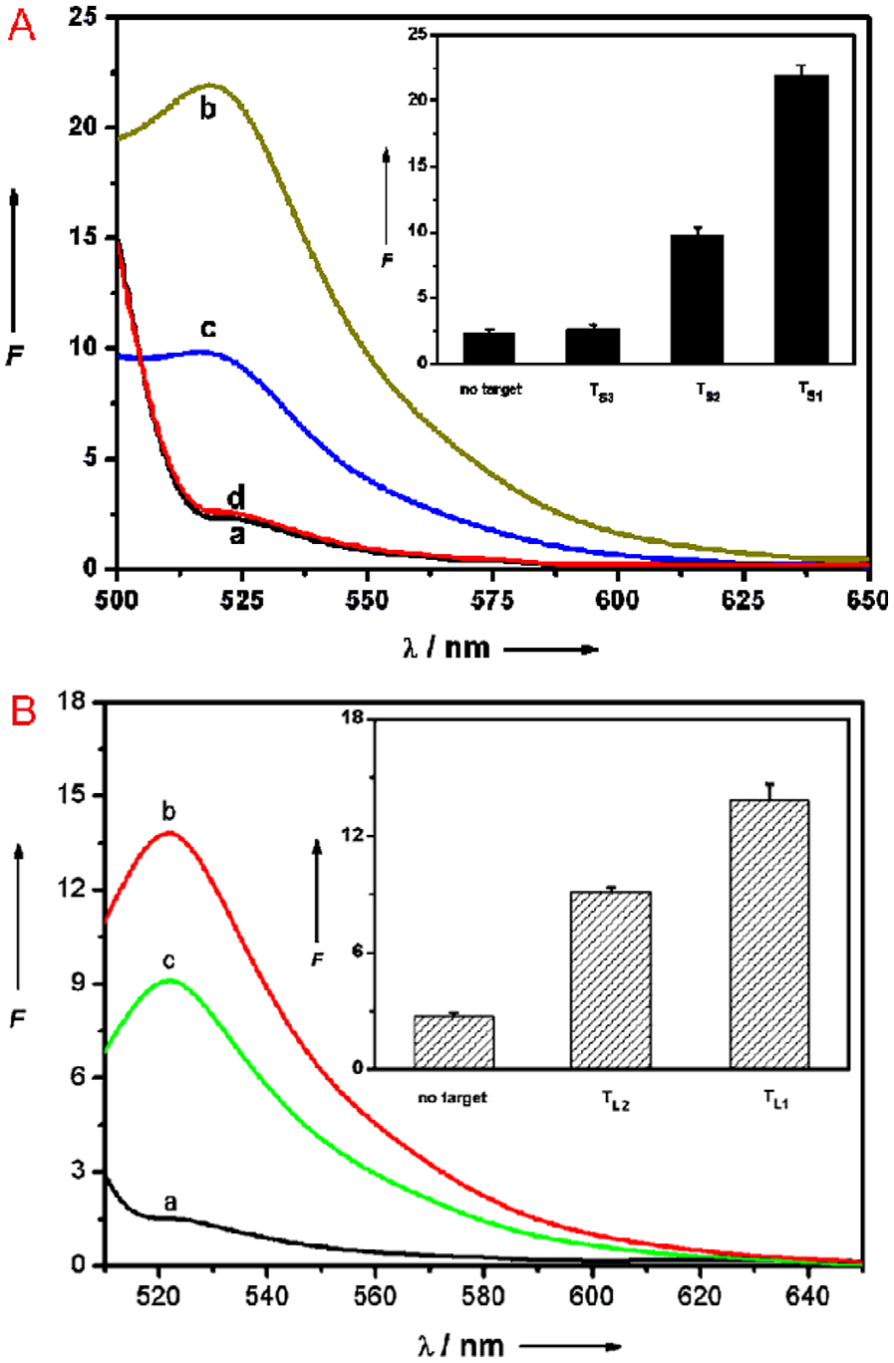
Evaluation of discrimination ability using (A) shorter probe or (B) longer target. (A) Fluorescence emission spectra of P_s_ (50 nM) at different conditions: (a) P_s_–PMPD complex; (b) P_s_–PMPD complex+300 nM T_s1_; (c) P_s_–PMPD complex+300 nM T_s2_; (d) P_s_–PMPD complex+300 nM T_s3_. (B) Fluorescence emission spectra of P_HIV_ (50 nM) in the presence of PMPD nanorods at different conditions: (a) P_HIV_–PMPD complex; (b) P_HIV_–PMPD complex+300 nM T_L1_; (c) P_HIV_–PMPD complex+300 nM T_L2_. Inset: fluorescence intensity histograms with error bar. Excitation was at 480 nm, and the emission was monitored at 522 nm. All measurements were done in Tris-HCl buffer in the presence of 5 mM Mg^2+^ (pH: 7.4). 10-µL PMPD nanorods were used in each measurement.

It is important to note that the use of shorter oligonucleotide can improve the mismatch discrimination ability of our present sensing system. Figure 7A shows the fluorescence responses of FAM-labeled, 9-nucleotide ssDNA probe P_s_ (50 nM) toward complementary target T_s1_, single-base mismatched target T_s2_, and non-complementary target T_s3_ at room temperature, in the presence of PMPD. The (*F*/*F*_0_–1) value obtained upon addition of 300 nM of T_s2_ is about 38% of the value obtained upon addition of 300 nM of T_s1_ into P_s_–PMPD complex. We further evaluated its ability of this sensing platform to distinguish single-base mismatch by mimicking the realistic situations, where a short oligonucleotide probe binds a small loci of a large DNA strand. Two long DNA strands were chosen as model systems: the middle part of T_L1_ is complementary target sequence to P_HIV_ and the middle part of T_L2_ is single-base-mismatched target sequence to P_HIV_. Figure 7B shows the fluorescence responses of P_HIV_ toward T_L1_ and T_L2_ in the presence of PMPD at room temperature. The addition of 300 nM of T_L1_ to P_HIV_–PMPD complex leads to about 41% fluorescence recovery which is much lower than 77% observed when 300 nM of T_1_ was used as the target. Such observation is not surprising given that P_HIV_–T_L1_ is a complex with a duplex DNA in the middle and two single strands on both ends and thus there are unpaired DNA bases for binding to PMPD. The (*F*/*F*_0_–1) value obtained upon addition of 300 nM of T_L2_ is about 58% of the value obtained upon addition of 300 nM of T_L1_ into P_HIV_–PMPD complex, indicating that this sensing platform is still able to discriminate complementary and mismatched target sequences embedded in a large DNA strand with a short oligonucleotide probe.

**Figure 8:**
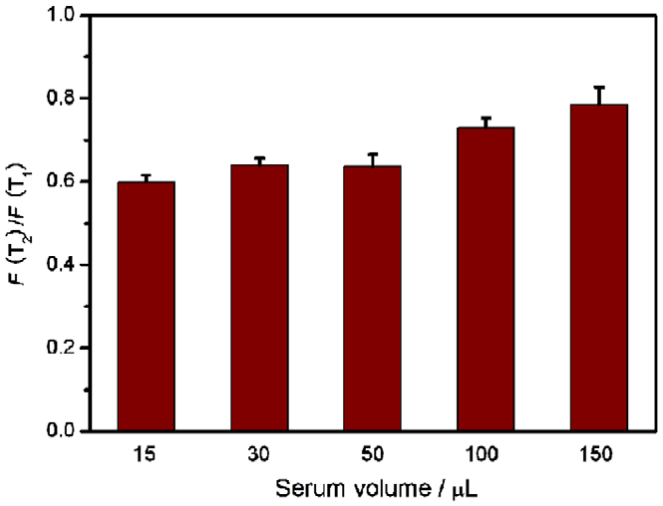
Performance of single-base mismatch discrimination in the presence of blood serum. The histograms of fluorescence intensity ratio of *F*(T_2_)/*F*(T_1_), where *F*(T_1_) and *F*(T_2_) are the fluorescence intensities in the presence of different amount of blood serum spiked with T_1_ and T_2_, respectively. ([T_1_] = 300 nM; [T_2_] = 300 nM). Excitation was at 480 nm, and the emission was monitored at 522 nm. The total volume of each sample was 300 µL in Tris-HCl buffer (pH 7.4) containing 5 mM Mg^2+^. 10-µL PMPD nanorods were used in each measurement.

We also performed DNA detection in human blood serum. Figure S6 shows the fluorescence emission spectra of P_HIV_ in the presence of 5% blood serum (volume ratio) in Tris-HCl buffer at different conditions. This system exhibits 86% fluorescence quenching and 50% fluorescence recovery and the difference of detection signal between single-base mismatched and complementary sequences is 40%. We further examined the influence of the amount of blood serum on the discrimination ability of this sensing system. Figure 8 shows the corresponding histograms of fluorescence intensity ratio of *F*(T_2_)/*F*(T_1_), where *F*(T_1_) and *F*(T_2_) are the fluorescence intensities in the presence of human blood serum spiked with T_1_ and T_2_, respectively. It suggests that the increase of blood serum in amount leads to decreased dicrimination ability. Note that, in the presence of different amount of blood serum, this system is still able to discriminate complementary and single-base mismatched sequences with good reproducibility. All the above observations indicate that there is no heavy interference from blood serum components on our measurements and thus this sensing system is promising for practically useful mismatch detection upon further development.

**Figure 9:**
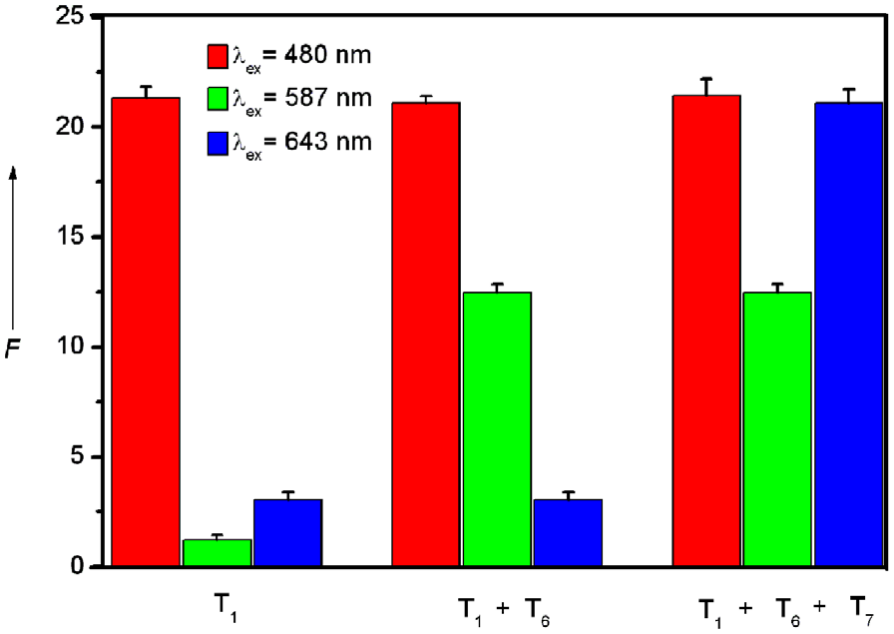
Multiplex DNA detection. Fluorescence intensity histograms of the probe mixture ([P_HIV_] = [P_HBV_] = [P_K167_] = 50 nM) toward different target combinations in the presence of PMPD nanorods under excitation/emission wavelengths of 480/522, 587/601, and 643/660 nm/nm. All measurements were done in Tris-HCl buffer in the presence of 5 mM Mg^2+^ (pH: 7.4). 10-µL PMPD nanorods were used in each measurement.

Multiplex detection of nucleic acid sequences is a challenge for many assays because of the need of eliminating probe set/target set cross-reactivity, minimizing nonspecific binding, and designing spectroscopically and chemically unique probes [57], which motivated us to explore the feasibility of using the platform described herein to detect multiple DNA targets simultaneously. To do this, we chose three probes (P_HIV_, P_HBV_, and P_K167_) labeled with FAM, ROX, and Cy5 (cyanine 5), respectively, as model systems. Because these three dyes are individually excited at 480, 587, and 643 nm to emit at 522, 601, and 660 nm, respectively, significant dye-to-dye energy transfer is avoided. It is found that the presence of PMPD leads to dramatic quenching of all dyes in the probe mixture, indicating that PMPD can effectively quench dyes of different emission frequencies. The absorption spectrum of the aqueous dispersion of PMPD nanorods shown in Figure S7 exhibits one strong peak at 206 nm and another pretty weak peak at 307 nm, suggesting there is no spectra overlap and thus no FRET occurs between PMPD and all the fluorescent dyes used. Figure 9 shows the fluorescence intensity histograms of the probe mixture toward different target combinations in the presence of PMPD under excitation/emission wavelengths of 480/522, 587/601, and 643/660 nm/nm, respectively. It is clearly seen that there is only one strong emission peak at 522 nm when excited at 480 nm in the presence of T_1_ only. However, the target combination of T_1_+T_6_ gives two strong emission peaks at 522 and 601 nm when excited at 480 and 587 nm, respectively. As expected, three strong emission peaks are observed for the T_1_+T_6_+T_7_ target combination at 522, 601, and 660 nm when excited at 480, 587, and 643 nm, respectively. Other target combinations also give similar results. All these observations indicate that this sensing platform can be used to detect multiple DNA targets with high selectivity.

In summary, PMPD nanospheres and nanorods can be selectively synthesized via the chemical oxidation polymerization of MPD monomers by APS with the use of different reaction solvent at room temperature. We demonstrate that such PMPD nanostructures can be used as an effective fluorescent sensing platform for multiplex nucleic acid detection with high sensitivity and selectivity. The fluorescence quenching mechanism invovled is also studied and the application of this sensing platform in human blood serum system is also demonstrated. Our present observations are significant for the following three reasons: (1) It provides us a facile method for the selective synthesis of PMPD nanostructures for multiplex and single-base mismatch detection of nucleic acid; (2) This PMPD-based assay holds great promise for practical application in clinical sample analysis; (3) It provides us a promising, universal and effective sensing platform for a fluorescence-enhanced detection sensitive and selective to the target molecule studied.

## Materials and Methods

All chemically synthesized oligonucleotides were purchased from Shanghai Sangon Biotechnology Co. Ltd. (Shanghai, China). DNA concentration was estimated by measuring the absorbance at 260 nm. All the other chemicals were purchased from Aladin Ltd. (Shanghai, China) and used as received without further purification. The water used throughout all experiments was purified through a Millipore system. The PMPD nanospheres (sample 1) were prepared as follows: In a typical experiment, 0.06 mL of 0.5 M APS aqueous solution was dilutted with 0.84 mL water at room temperature, followed by the addition of 0.1 mL of 0.1 M MPD aqueous solution under shaking. After that, a large amount of precipitates were observed. The resulting precipitates were washed with water by centrifugation twice first, and then redispersed in water to give a 8.6-µg/mL suspension and stored at 4°C for characterization and further use. The PMPD nanorods were similarly prepared except that an equivalent aliquot of N-methylpyrrolidone (NMPD) was substituted for the 0.84 mL water. The volume of each sample for fluorescence measurement is 300 µL in 20 mM Tris-HCl buffer containing 100 mM NaCl, 5 mM KCl, and 5 mM MgCl_2_ (pH: 7.4).

The experiments of multiplex detection were performed as follows: In a typical multiplex assay, P_HIV_, P_HBV_, and P_K167_ were added into Tris-HCl buffer (containing 20 mM Tris-HCl, 100 mM NaCl, 5 mM KCl and 5 mM MgCl_2_, pH 7.4) to give a mixture of probes ([P_HIV_] = [P_HBV_] = [P_K167_] = 50 nM). After that, PMPD nanorods were added and the resultant mixture was incubated over a 1-h period, and then the fluorescence emission spectra were collected. Different target combinations were then added and the fluorescence emission spectra were collected after incubation over a 1-h period.

Scanning electron microscopy (SEM) measurements were made on a XL30 ESEM FEG scanning electron microscope at an accelerating voltage of 20 kV. Fluorescent emission spectra were recorded on a PerkinElmer LS55 Luminescence Spectrometer (PerkinElmer Instruments, U.K.). Zeta potential measurement was performed on a Nano-ZS Zetasizer ZEN3600 (Malvern Instruments Ltd., U.K.). An energy-dispersive X-ray spectroscopic detecting unit was used to collect the energy-dispersed spectrum (EDS) for elemental analysis.

Oligonucleotide sequences are listed as follows (mismatch underlined):

[1] P_HIV_ (FAM dye-labeled ssDNA):

5′-FAM-AGT CAG TGT GGA AAA TCT CTA GC-3′

[2] T_1_ (complementary target to P_HIV_):

5′-GCT AGA GAT TTT CCA CAC TGA CT-3′

[3] T_2_ (single-base mismatched target to P_HIV_):

5′-GCT AGA GAT TGT CCA CAC TGA CT-3′

[4] T_3_ (two-base mismatched target to P_HIV_):

5′-GCT AGA GAT TGT ACA CAC TGA CT-3′

[5] T_4_ (three-base mismatched target to P_HIV_):

5′-GCT ATA GAT TGT ACA CAC TGA CT-3′

[6] T_5_ (non-complementary target to P_HIV_):

5′-TTT TTT TTT TTT TTT TTT TTT TT-3′

[7] P_s_ (FAM dye-labeled shorter ssDNA):

5′-TGG AAA ATC-3′

[8] T_s1_ (complementary target to P_s_):

5′-GAT TTT CCA-3′

[9] T_s2_ (single-base mismatched target to P_s_):

5′-GAT TGT CCA-3′

[10] T_s3_ (non-complementary target to P_s_):

5′-TTT TTT TTT-3′

[11] P_HBV_ (ROX dye-labeled ssDNA):

5′-ROX-TAC CAC ATC ATC CAT ATA ACT GA-3′

[12] T_6_ (complementary target to P_HBV_):

5′-TCA GTT ATA TGG ATG ATG TGG TA-3′

[13] P_K167_ (Cy5 dye-labeled ssDNA):

5′-Cy5-TCT GCA CAC CTC TTG ACA CTC CG-3′

[14] T_7_ (complementary target to P_K167_):

5′-CGG AGT GTC AAG AGG TGT GCA GA-3′

[15] T_L1_ (The middle part of a long strand as a target complementary to P_HIV_):

5′-TTT TTT TTT TTT TTT TTT TTT TGC TAG AGA TTT TCC ACA CTG ACT TTT TTT TTT TTT TTT TTT TTT T-3′

[16] T_L2_ (The middle part of a long strand as a single-base mismatched target to P_HIV_):

5′-TTT TTT TTT TTT TTT TTT TTT TGC TAG AGA TTG TCC ACA CTG ACT TTT TTT TTT TTT TTT TTT TTT T-3′

## Supporting Information

Figure S1 **Chemical composition analysis.** EDS of the PMPD nanospheres thus formed.
(TIF)

Figure S2 **PMPD formation under different conditions.** SEM images of the PMPD nanostructures formed using (a) DMF, (b) ethanol as the reaction solvent, and (c) water as solvent at basic condition by adding NH_3_·H_2_O.
(TIF)

Figure S3 **Adsorption of P_HIV_ on PMPD confirmation.** Fluorescence spectra of (a) P_HIV_–PMPD complex+T_1_ and (b) the supernatant of (a) after removing PMPD by centrifugation. ([P_HIV_] = 50 nM; [T_1_] = 300 nM; *λ*_ex_ = 480 nm). All measurements were done in Tris-HCl buffer in the presence of 5 mM Mg^2+^ (pH: 7.4). The concentration of PMPD nanorods used is 8.5 mg/mL and an optimal volume of 10 µL was chosen in our present study.
(TIF)

Figure S4 **Investigation of the influence of the amount of PMPD on the system.** Fluorescence intensity histograms of P_HIV_+PMPD and P_HIV_+PMPD+T_1_ with the use of 0, 5, 10, 15, and 20-µL PMPD sample in this system, respectively. ([P_HIV_] = 50 nM; [T_1_] = 300 nM; λ_ex_ = 480 nm). The concentration of PMPD nanorods is 8.5 mg/mL.
(TIF)

Figure S5 **Stern–Volmer quenching constant (K_SV_) determination.** Stern–Volmer quenching curve describing *F_0_*/*F* as a function of MPD concentration, where *F*_0_ and *F* are FAM fluorescence intensities at 522 nm in the absence and the presence of PMPD nanorods, respectively ([P_HIV_] = 50 nM).
(TIF)

Figure S6 **Performance of DNA detection in the presence of blood serum.** Fluorescence emission spectra of P_HIV_ (50 nM) at different conditions: (a) blank; (b) P_HIV_; (c) P_HIV_+300 nM T_1_; (d) P_HIV_+PMPD nanorods; (e) P_HIV_+PMPD nanorods+300 nM T_1_; (f) P_HIV_+PMPD nanorods+300 nM T_2_. Excitation was at 480 nm, and the emission was monitored at 522 nm. All measurements were done in Tris-HCl buffer in the presence of 5% blood serum (volume ratio) and 5 mM Mg^2+^ (pH: 7.4).
(TIF)

Figure S7 **UV-Vis absorption of PMPD.** Absorption spectrum of PMPD nanorods dispersed in Tris-HCl buffer in the presence of 5 mM Mg^2+^ (pH 7.4).
(TIF)
