# Supplementary figures and images for: Poly(m-Phenylenediamine) Nanospheres and Nanorods: Selective Synthesis and Their Application for Multiplex Nucleic Acid Detection
Source: PLoS One. 2011 Jun 23;6(6):e20569. doi: 10.1371/journal.pone.0020569 (PMC3121721; doi:10.1371/journal.pone.0020569)

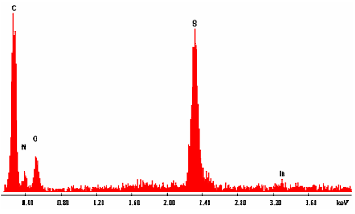

Supplement: Figure S1 — Chemical composition analysis. EDS of the PMPD nanospheres thus formed. (TIF) [file pone.0020569.s001.tif]

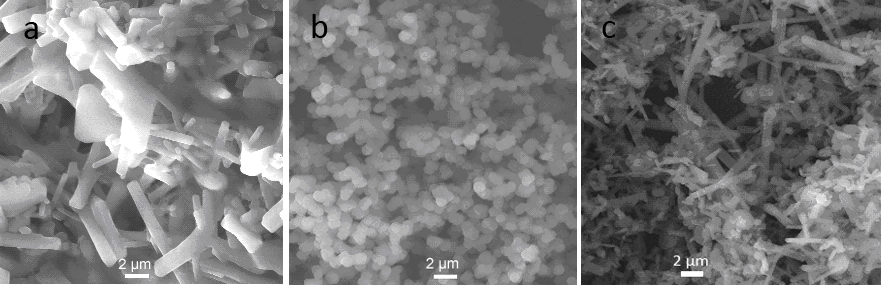

Supplement: Figure S2 — PMPD formation under different conditions. SEM images of the PMPD nanostructures formed using (a) DMF, (b) ethanol as the reaction solvent, and (c) water as solvent at basic condition by adding NH3·H2O. (TIF) [file pone.0020569.s002.tif]

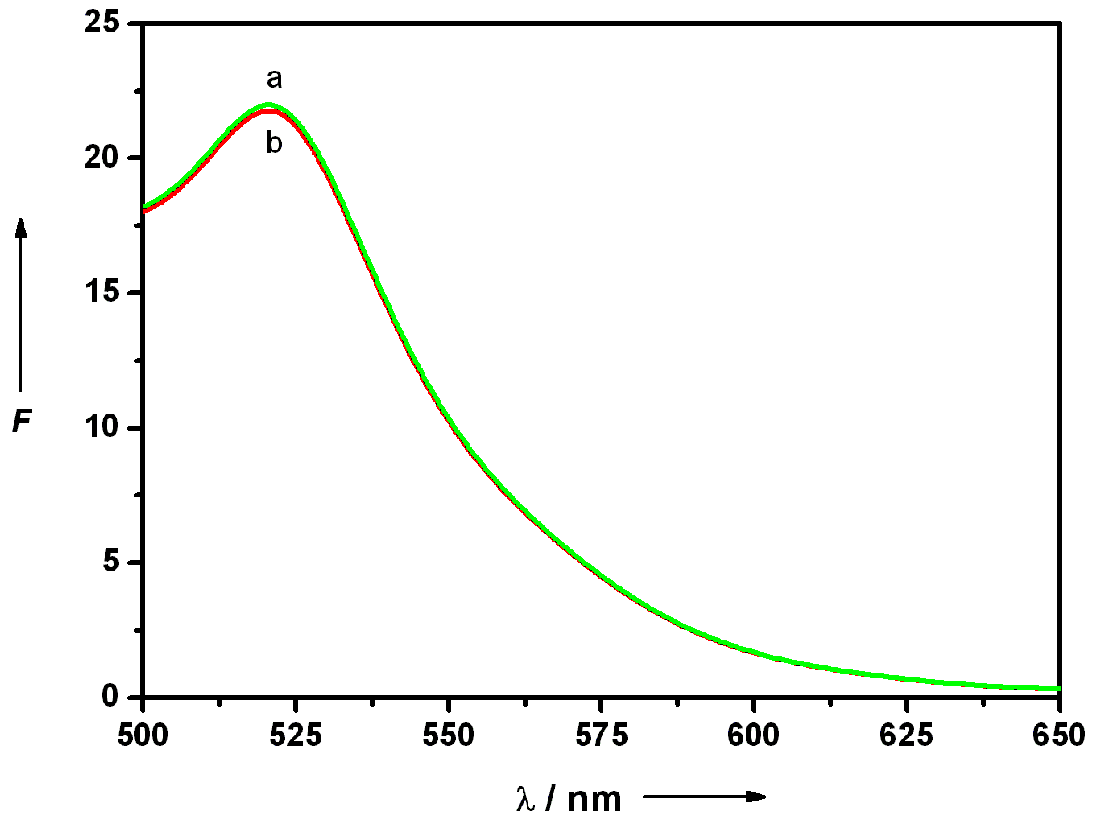

Supplement: Figure S3 — Adsorption of PHIV on PMPD confirmation. Fluorescence spectra of (a) PHIV–PMPD complex+T1 and (b) the supernatant of (a) after removing PMPD by centrifugation. ([PHIV] = 50 nM; [T1] = 300 nM; λ ex = 480 nm). All measurements were done in Tris-HCl buffer in the presence of 5 mM Mg2+ (pH: 7.4). The concentration of PMPD nanorods used is 8.5 mg/mL and an optimal volume of 10 µL was chosen in our present study. (TIF) [file pone.0020569.s003.tif]

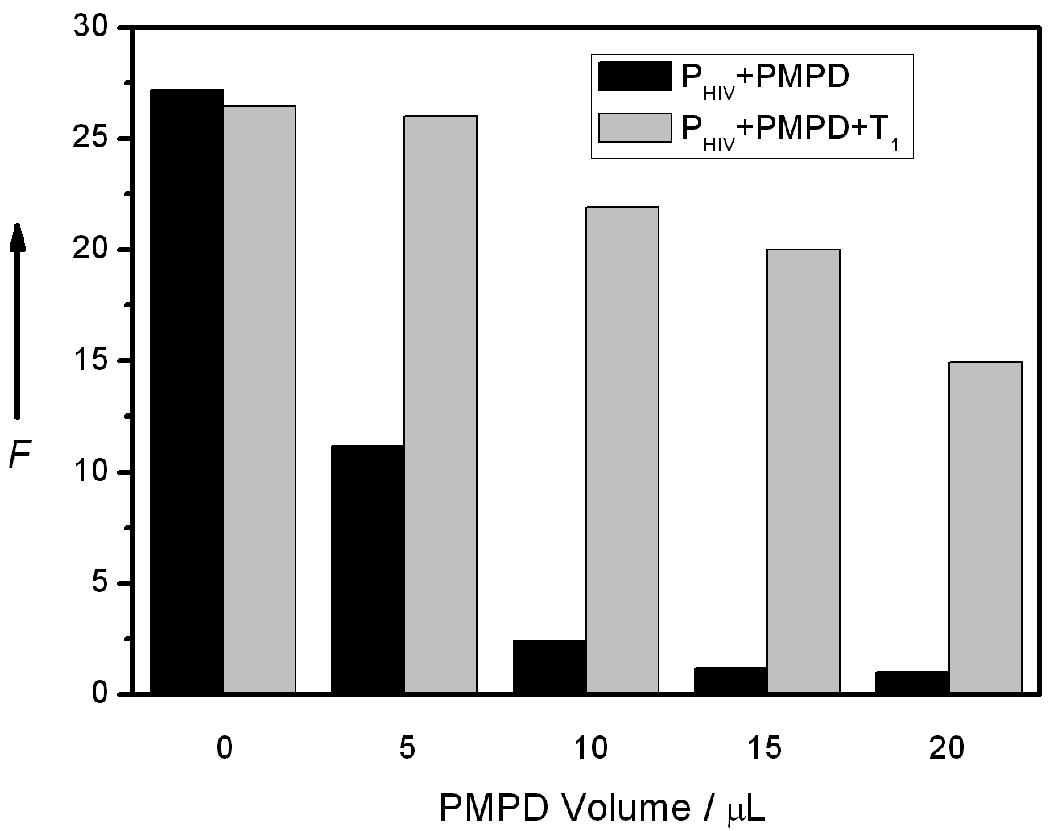

Supplement: Figure S4 — Investigation of the influence of the amount of PMPD on the system. Fluorescence intensity histograms of PHIV+PMPD and PHIV+PMPD+T1 with the use of 0, 5, 10, 15, and 20-µL PMPD sample in this system, respectively. ([PHIV] = 50 nM; [T1] = 300 nM; λex = 480 nm). The concentration of PMPD nanorods is 8.5 mg/mL. (TIF) [file pone.0020569.s004.tif]

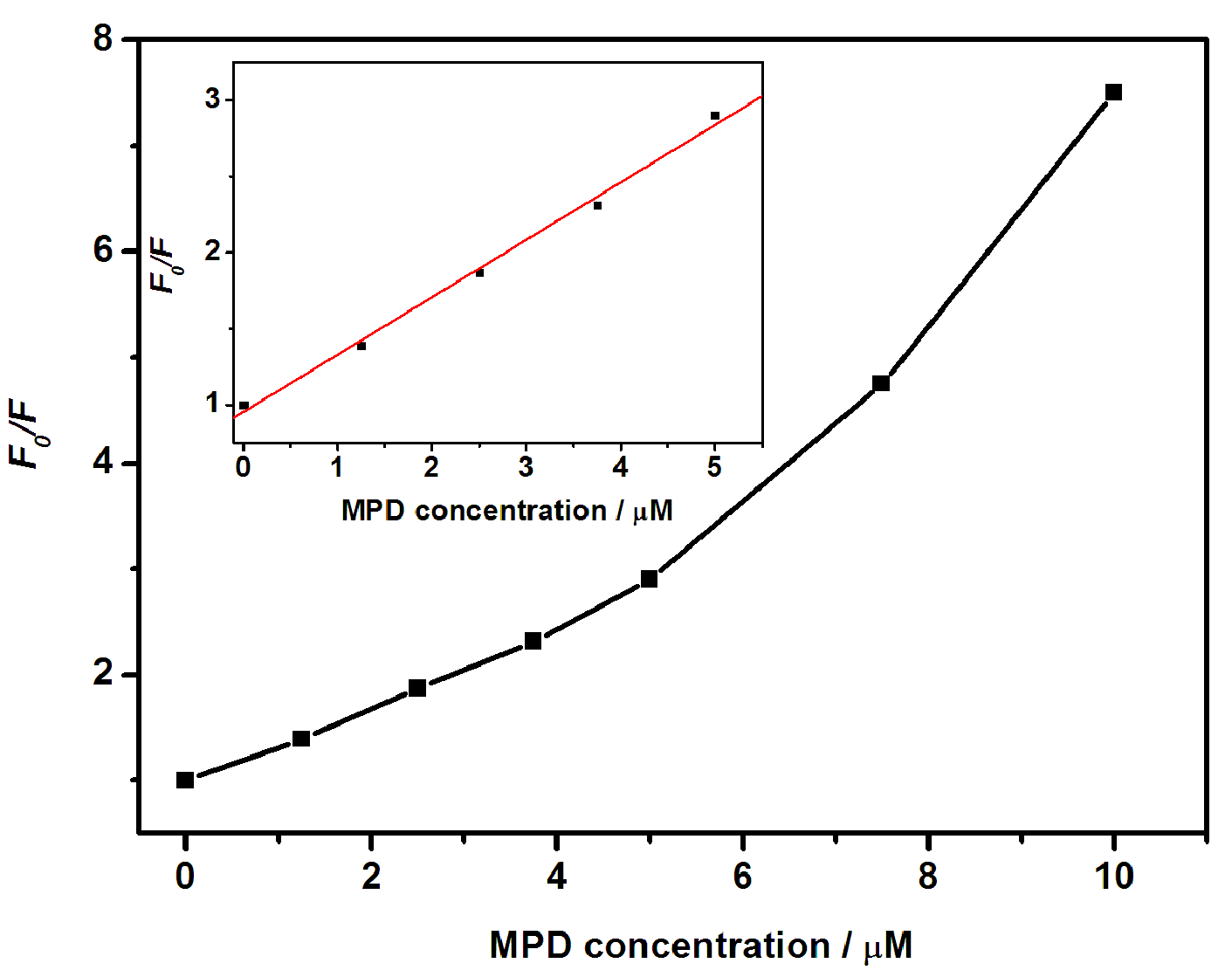

Supplement: Figure S5 — Stern–Volmer quenching constant (KSV) determination. Stern–Volmer quenching curve describing F0/F as a function of MPD concentration, where F 0 and F are FAM fluorescence intensities at 522 nm in the absence and the presence of PMPD nanorods, respectively ([PHIV] = 50 nM). (TIF) [file pone.0020569.s005.tif]

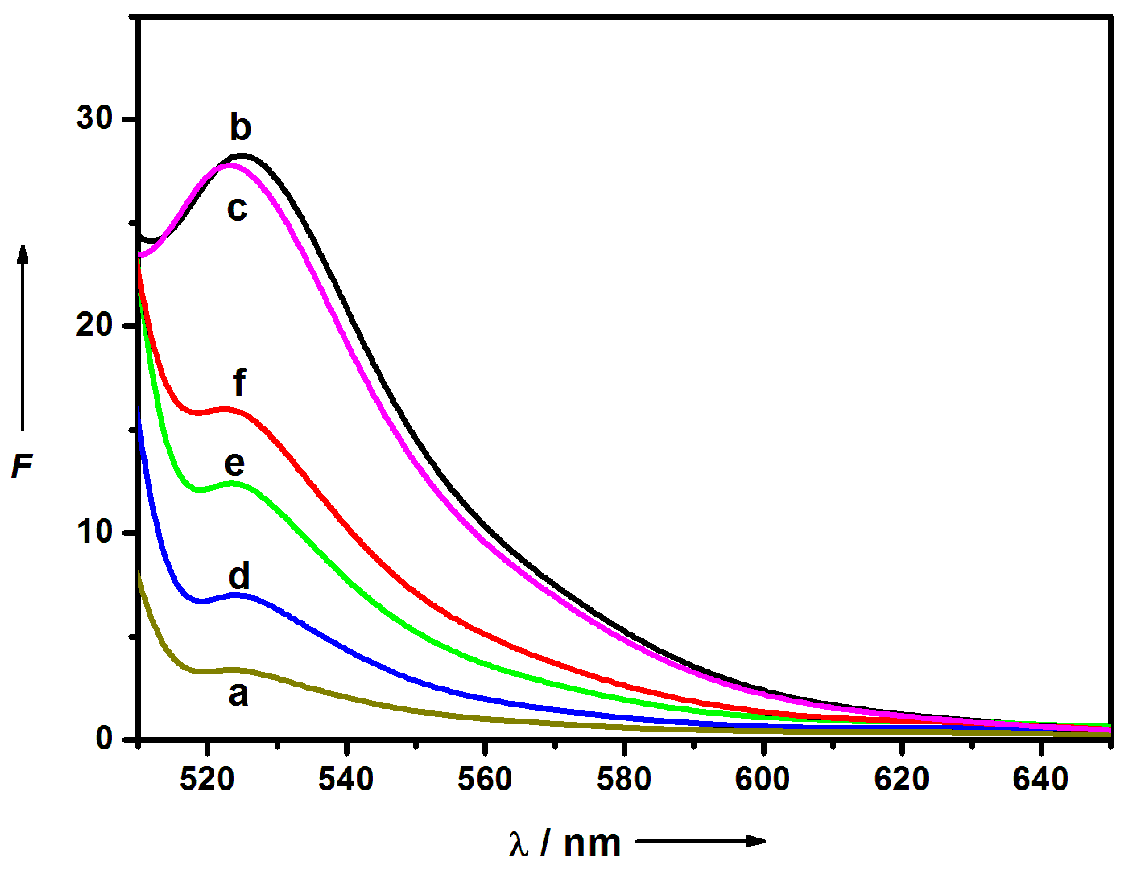

Supplement: Figure S6 — Performance of DNA detection in the presence of blood serum. Fluorescence emission spectra of PHIV (50 nM) at different conditions: (a) blank; (b) PHIV; (c) PHIV+300 nM T1; (d) PHIV+PMPD nanorods; (e) PHIV+PMPD nanorods+300 nM T1; (f) PHIV+PMPD nanorods+300 nM T2. Excitation was at 480 nm, and the emission was monitored at 522 nm. All measurements were done in Tris-HCl buffer in the presence of 5% blood serum (volume ratio) and 5 mM Mg2+ (pH: 7.4). (TIF) [file pone.0020569.s006.tif]

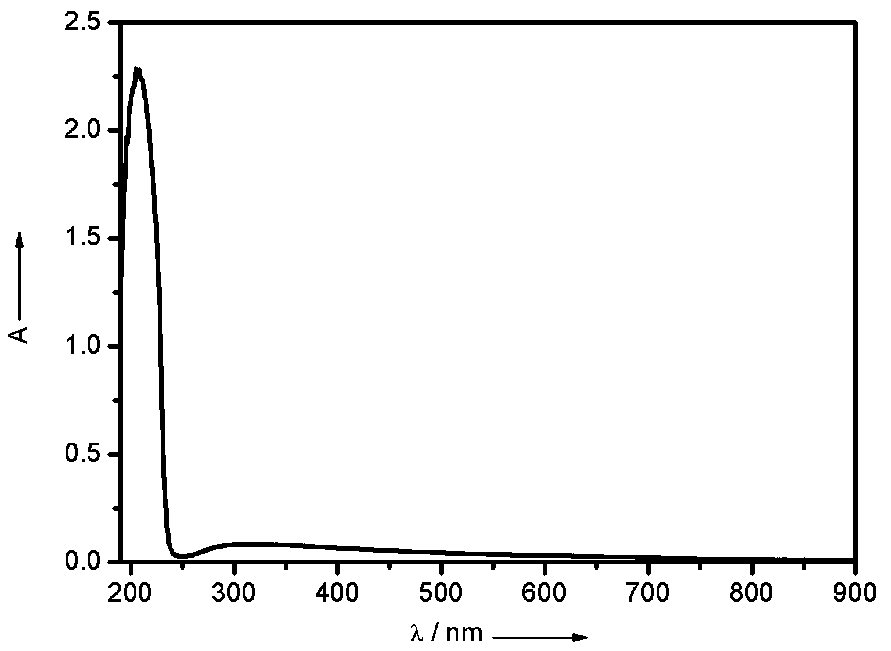

Supplement: Figure S7 — UV-Vis absorption of PMPD. Absorption spectrum of PMPD nanorods dispersed in Tris-HCl buffer in the presence of 5 mM Mg2+ (pH 7.4). (TIF) [file pone.0020569.s007.tif]
